# The effects of rhythmic auditory stimulation on functional ambulation after stroke: a systematic review

**DOI:** 10.1186/s12906-023-04310-3

**Published:** 2024-01-20

**Authors:** Samira Gonzalez-Hoelling, Gloria Reig-García, Carme Bertran-Noguer, Rosa Suñer-Soler

**Affiliations:** 1 Neurorehabilitation department, Hospital Sociosanitari Mutuam Girona, 17007 Girona, Catalonia Spain; 2 Department of Nursing, Faculty of Nursing, University of Girona, 17003 Girona, Spain; 3 Health and Health Care Research Group, University of Girona, 17003 Girona, Spain

**Keywords:** Rehabilitation, Music therapy, Walking ability, Walking aids

## Abstract

**Background:**

Several studies have reported the effect of rhythmic auditory stimulation (RAS) on functional ambulation in stroke patients, yet no systematic overview has yet been published. This study aims to synthesize the available evidence describing changes in stroke patients after RAS intervention for functional ambulation and the use of walking assistive devices, and to find out if the effect of RAS and music-based RAS differs depending on the lesioned area.

**Methods:**

The PubMed, PEDro, Cochrane Central Register of Controlled Trials, Web of Science, Scopus and CINAHL electronic databases were searched for reports evaluating the effect of RAS on walking in stroke patients, applying the PICOS criteria for the inclusion of studies.

**Results:**

Twenty one articles were included (948 stroke survivors). Most studies were of good methodological quality according to the PEDro scale, but they had a high risk of bias. The most consistent finding was that RAS improves walking and balance parameters in stroke patients in all phases compared to baseline and versus control groups with conventional treatment. Functional ambulation and the use of walking assistive devices were inconsistently reported. Several studies also suggest that RAS may be as good as other complementary therapies (horse-riding and visual cueing).

**Conclusions:**

Despite the beneficial effects of RAS, the question remains as to whether it is better than other complementary therapies. Given the heterogeneity of the interventions, the interventions in control groups, the varied durations, and the different outcome measures, we suggest that care should be taken in interpreting and generalizing findings.

**PROSPERO Registration:**

CRD42021277940.

**Supplementary Information:**

The online version contains supplementary material available at 10.1186/s12906-023-04310-3.

## Background

Stroke is the consequence of an alteration in cerebral circulation that causes a transitory or permanent deficit in the functioning of one or more areas of the brain. It is a leading cause of adult and motor function impairments [1].

Walking ability is one of the most affected functions in patients with stroke. Physical rehabilitation is effective for recovery of function and mobility after stroke but no single approach seems to be more or less effective than others [2]. Motor control during ambulation is composed of balance control, muscle activation and task-oriented movement [3]. The disruption of descending neural pathways is common in hemiparesis after stroke, even if there is no lesion of the motor automatic process structures such as the cerebellar or the brainstem. Walking speed after a rehabilitation process is associated with community mobility and higher scores in quality of life [4]. Walking speed improves with different rehabilitation approaches but asymmetric postural behaviour is maintained or reinforced by the predominant use of the ipsilesional side with walking assistive devices [3]. Tyson and Rogerson (2009) showed that the use of assistive devices in early stroke improved functional ambulation but did not find any differences in walking ability (speed and step length). Participants in this study preferred to walk with the devices rather than delay walking until ambulation was reached without them [5].

Rhythmic auditory stimulation (RAS) is a neurologic music therapy (NMT) rehabilitation technique that has been adopted since 2019 in the Canadian Stroke Care Guidelines [6]. This therapy involves the presentation of auditory rhythmic cues in the form of repetitive isochronous pulses (e.g., metronome clicks) or metrically accentuated music with an embedded metronome to promote auditory-motor entrainment of intrinsically rhythmic movements[7]. In the 1990s researchers showed how musical-rhythmic stimuli can improve mobility, executive functions and other cognitive functions such as attention and memory in stroke and Parkinson’s disease [6–8].

Over the last twenty years, researchers have found that people with neurological disorders such as stroke could improve motor control, movement and re-programme the execution of movement patterns with musical rhythm entrainment [7]. Systematic reviews have shown that rhythmic auditory stimulation interventions have beneficial effects on gait velocity, stride length, cadence, and postural stability [7, 9–12]. Janzen et al. (2022) suggest that clinical stage, cognitive capacity and motor function at enrolment should be considered for a feasible and effective intervention in stroke [7].

A large number of publications have addressed rhythmic auditory stimulation in stroke, including Wang et al. (2022), who recently undertook a systematic review about motor function and balance ability. To date no systematic review has been conducted that synthesises the scientific evidence about the effects of RAS or music-based rhythmic auditory stimulation on the use of assistive devices (functional ambulation) in patients with stroke, and neither has any review examined the effectiveness of RAS or music-based RAS effectiveness in relation to the lesioned area. The current systematic review aimed to: (1) collect and critically appraise all the available evidence about the effects of RAS and music-based RAS for functional ambulation and the use of walking assistive devices; and (2) find out whether the effect of RAS and music-based RAS differs depending on the lesioned area.

## Methods

### Information sources

A comprehensive search of PubMed, PEDro, Cochrane Central Register of Controlled Trials, Web of Science, Scopus, CINAHL, was done with the latest search performed on 15th November 2023. The following MeSH terms have been used in conducting the search: (“rhythmic auditory stimulation”) AND (“stroke” OR “cerebrovascular disease”) AND (“rehabilitation”), more details of all databases can be found in Supplementary Table 1. The search was further limited to the English language and human subjects. We identified both randomised controlled trials and quasi-experimental trials of rhythmic auditory stimulation for walking ability and the use of walking assistive devices in post-stroke patients, from January 2012 to November 2023. Reference lists of potentially eligible studies were hand searched.

The study protocol was registered in PROSPERO (Prospective International Register of Systematic Reviews; registration number CRD42021277940), and was developed following the guidelines of Preferred Reporting Items for Systematic Reviews and Meta-analysis Protocols (PRISMA-P) [13], which is available as supplementary Table 2. Studies were included if they met the PICOS criteria.

### Eligibility criteria

#### Types of studies

We included randomized controlled trials (RCTs) which were more likely to provide unbiased information than other study designs, but also quasi-experimental clinical trials. Cross-over trials were excluded because of potential carry-over effects. Only full-text studies written in English, Spanish, German, French or Portuguese were considered for inclusion.

#### Types of participants

We included adults (over 18 years old) with balance and gait disorders after stroke. No limitations were set on stroke type (ischaemic or haemorrhagic) or phase (acute, subacute or chronic). Acute and subacute stroke is defined as less than 6 months since onset, and chronic stroke lasts more than 6 months since onset. There were no restrictions on the sex or ethnicity of the enrolled subjects.

#### Types of interventions

##### Experimental interventions

Trials using either rhythmic auditory stimulation or music-based rhythmic auditory stimulation were included. Rhythmic auditory stimulation refers to a neurologic music therapy (NMT) rehabilitation technique that involves the presentation of auditory rhythmic cues in the form of repetitive isochronous pulses (e.g., metronome clicks) or metrically accentuated music with an embedded metronome. Music-based rhythmic auditory stimulation is the use of auditory rhythmic cues in the form of metrically accentuated music with or without metronome [7]. We excluded trials with other music techniques, such as playing instruments, listening to music and dancing to music. No restrictions were imposed on the duration of the treatment sessions nor on the length of the treatment period.

##### Comparator interventions

The control interventions were either physical therapy or another complementary active treatment. Physical therapy is defined as repetitive task training, caregiver-mediated exercise, and circuit class therapy on balance and postural control. We considered other complementary active treatments, such as horse riding, yoga, water-based exercises, and others.

#### Types of outcome measures

##### Primary outcome

The primary outcome measure was functional ambulation ability and the use of walking assistive devices. Walking ability or functional ambulation ability is defined as the ability to walk, with or without the aid of appropriate assistive devices, safely and sufficiently to carry out mobility-related activities of daily living. Walking ability includes the effects of multiple systems (e.g., cognition, balance, motor, perception), which is why walking ability may be a significant marker for global health ratings. The functional ambulation category was used as an evaluation measure for walking ability. A walking assistive device is defined as a piece of equipment that helps people to move independently and includes walkers, crutches, and canes.

##### Secondary outcome

Secondary outcome measures include functional independence, falls and quality of life. Functional independence was measured based on scales such as the Functional Independence Measure (FIM), the Barthel Index, and the modified Rankin scale. A fall is defined as ﻿an event reported by the faller or a witness, resulting in a person inadvertently coming to rest on the ground or another lower level, with or without loss of consciousness or injury. Quality of life was measured by generic or condition-specific scales, such as the Short Form 36 Health Survey.

### Data extraction

#### Selection process

The selection process for the included studies was carried out in three phases by four researchers. In the case of discrepancies, these were discussed and a consensus was reached. Firstly, Mendeley bibliographic management software was used to compile the bibliographic references for each of the included databases, to eliminate duplicates, and to screen and select studies to be included in the systematic review. Secondly, once duplicates had been removed, the titles and abstracts of all studies identified by the search strategy were examined, and non-relevant studies were excluded. Thirdly, the full texts of studies to be included in the second screening phase were obtained and the eligibility criteria were assessed. Any disagreement about the eligibility of particular studies were resolved by discussion between all four researchers.

In the same way, the reference lists of identified and included studies were examined (advanced search) in order to identify any study not filtered by the search equation. One last search was conducted before the final submission of the manuscript.

#### Extraction and qualitative synthesis of the data

Four authors independently extracted data from the selected trials using a standardised coding form. Any differences in data extraction were discussed. Data were recorded in two separate Excel spreadsheets, one for controlled-trials and the other for quasi-experimental trials, specifically designed to facilitate the selection process, data extraction and analysis, including (1) first author, (2) year of publication, (3) sample size, (4) randomization (5) blinding, (6) stroke type, (7) stroke brain side—hemisphere and area, (8) stroke phase, (9) intervention used, (10) treatment in the control or comparative group, (11) setting (12) outcomes, (13) measures, (14) adverse effects, (15) main results, (16) time of intervention, and (17) use of walking assistive devices.

A narrative synthesis was used to describe and compare the studies (quantitative synthesis regarding measures of central tendency, risk coefficient, 95% confidence intervals and the level of statistical significance of the p-value, as appropriate). There were no minimum number of studies required for synthesis. Considering the inconsistency of the assessment tools, data consolidation was used to describe the main results. The main quality variables and study results are summarized and tabulated.

#### Risk of bias (quality) assessment

Four review authors independently assessed all included trials for trial quality. We used the Cochrane Collaboration's Risk of Bias tool in the Cochrane Manual V.5.1.0 [14] to evaluate the risk of bias of each included study. The contents include random sequence generation, allocation sequence concealment, incomplete outcome data, blinded outcome assessment, selective outcome reporting, and other sources of bias. Blinding as a characteristic was not possible given the specific intervention that is being investigated. The assessment results were divided into three levels: low risk, high risk and uncertain risk. For the controlled trials, the PEDro scale was used to quality assessment; this includes 11 items such as random allocation of the subjects, concealed allocation, and the blinding of therapists and assessors. The total PEDro score was considered of good quality when it was six or higher [15].

## Results

### Study selection process

**Fig. 1:**
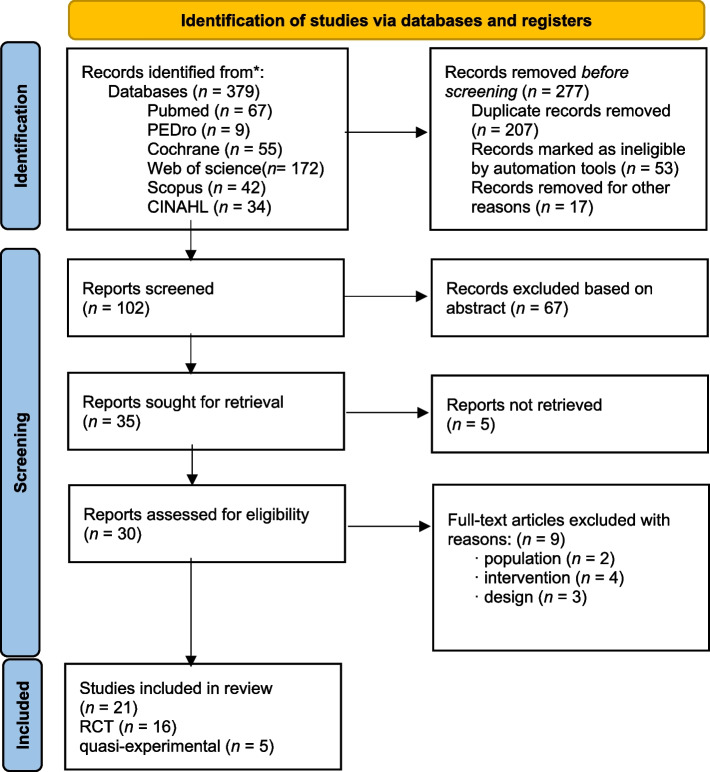
PRISMA-compliant flowchart

Our database search yielded 379 titles of which 21 were finally included [16–36]. Figure 1 contains a PRISMA-compliant flowchart of search results and selection for the review. Overall, five discrepancies were found between the abstraction forms completed by the four independent reviewers; these were resolved via discussion. Meta-analysis could not be conducted due to the heterogeneity of the data. Subgroup analysis was not performed due to lack of data. Sixteen studies were controlled trials, and the remaining studies were characterised as non-controlled trials. Most studies did not meet the criteria of blinding the subjects and therapist, as this would not have been possible given the nature of the treatments.

### Studies included

Study designs included fifteen randomized controlled trial, one non-randomized controlled trial and five pre-post studies. In nine studies, the assessors were blinded to treatment allocation. The sources of bias included sampling, selection, performance and measurement biases (Supplementary Fig. 1). A quality assessment of the included studies using the PEDro checklist can be found in Supplementary Table 3. Overall, the included studies scored high on the PEDro scale. The items in the PEDro allocated for eligibility criteria and the random allocation of subjects was high across studies. Given the logical absence of patient and therapist blinded studies, no scores could be calculated for this item. Subject allocation concealment and the blinding of assessors were often scored low, as this information was mostly missing. Three studies gave no information regarding between-group changes and comparison. As a result, internal validity mostly scored low.

The sample sizes ranged from 10 to 123 people diagnosed with stroke, and a total of 948 participants were included: detailed information about participants characteristics can be found in Table 1.

**Table 1:** Participant characteristics of all studies

Study	n RAS / control	Stroke type H: Isch RAS / control (/control)	Stroke side R/L (vascular area)	Stage of stroke	Time since onset (months) RAS/control	Setting
Ahmed et al., 2023 [36]	30 15/15	Not specified	Not specified	Chronic	15.83 ± 3.90/16.66 ± 4.44	Rehabilitation centre
Bunketorp-Käll et al., 2017 [16]	123 41/41/41	9:32/13:28/14:27	58/64	Chronic	32.33 ± 14.10/36.54 ± 14.63/36.73 ± 19.20	Rehabilitation centre
Bunketorp-Käll et al., 2019 [17]	123	9:32/13:28/14:27	58/64	Chronic	32.33 ± 14.10/ 36.54 ± 14.63/36.73 ± 19.20	Rehabilitation centre
Cha Y. et al., 2014 [18]	41/0	0:41	22/19	Chronic	8.68 ± 2.35	Rehabilitation centre
Cha Y. et al., 2014 [19]	20 10/10	0:10/0:10	2/18	Chronic	14.5 ± 5.5/14.7 ± 5.4	Rehabilitation centre
Cho et al., 2020 [20]	30 15/15	8:7/6:9	18/12	Chronic	30.33 ± 7.69/26.13 ± 6.58	Rehabilitation centre
Choi et al., 2021 [21]	16 8/8	Not specified	Not specified	Not specified	Not specified	Rehabilitation hospital
Chouhan et al., 2012 [22]	45 15/15/15	Not specified	45 MCA	Not specified	Not specified	Rehabilitation hospital
Collimore A. et al., 2023	10/0	Not specified	6/4	Chronic	> 6 months	Not specified
Elsner et al., 2019 [23]	12 6/6	0:6/0:6	12/0	Chronic	34.7 ± 20.1/99.2 ± 88.5	Rehabilitation centre
Gonzalez-Hoelling et al., 2021 [24]	55 28/27	9:17/9:19	37/16 12 basal ganglia 13 MCA 10 vertebrobasilar 9 lacunar 5 cerebellar 1 MCA + ACA 1 thalamus	Subacute	0.34 ± 0.13/﻿0.33 ± 0.12	Rehabilitation hospital
Hutchinson et al., 2020 [25]	11 11/0	Not specified	6/5	Chronic	> 6 months	Not specified
Kim et al., 2012 [26]	20 10/10	4:6/8:2	12/8	Chronic	5.7/4.8	Rehabilitation centre
Ko et al., 2016 [27]	15/0	Not specified	7/8	Chronic	81.9 ± 87.8	Rehabilitation centre
Kobinata et al., 2016 [28]	105/0	48:57	20 cerebellum 26 pons and medulla 22 thalamus 18 putamen 19 corona radiata	Subacute	1.46 ± 0.90	Rehabilitation centre
Lee et al., 2018 [29]	44 23/21	4:19/3:18	15 /28 MCA 37, ACA 7	Chronic	34.7 ± 20.1/99.2 ± 88.5	Rehabilitation centre
Mainka et al., 2018 [30]	45 15/15/15	Not specified	20/15 17 MCA 10 brain stem, 4 thalamus/ basal ganglia 2 internal capsule 1 ACA 1 PCA	Subacute	RAS TT ﻿1.40 ± 0.99/TT 1.54 ± 0.77/NDT 1.18 ± 0.55	Rehabilitation hospital
Song et al., 2016 [31]	40 20/20	Not specified	17/23	Chronic	12.30 ± 3.4/14.75 ± 6.9	Rehabilitation hospital
Suh et al., 2014 [32]	16 8/8	3:5/2:6	6/10	Chronic	12.70 ± 9.31/7.37 ± 7.0	Rehabilitation centre
Wang Y et al., 2021 [33]	60	0:30/0:30	28/32	Subacute + Chronic	8.39 ± 2.09/8.45 ± 2.11	Rehabilitation hospital
Yang et al., 2016 [34]	22 11/11	7:4/6:5	13/9	Chronic	11.18 ± 3.68/11.97 ± 3.53	Rehabilitation hospital

*MCA* middle cerebral artery, *ACA* anterior cerebral artery, *PCA* Posterior cerebral artery, *RAS* Rhythmic auditory stimulation, *TT* Treadmill training, *NDT* Neurodevelopmental therapy

Five studies were of subacute stroke patients [22, 24, 28, 30, 33] while the others were of chronic phase patients, except for one study that did not inform about the time since stroke onset [21]. Most studies had patients with both ischaemic and haemorrhagic strokes, but seven studies did not provide information about the type of stroke [21, 22, 27, 30, 31, 35, 36]. The stroke hemisphere was not described in three studies [21, 22, 36], and the stroke area was analysed in four studies [24, 28–30]. The settings of the interventions were in rehabilitation hospitals on an inpatient basis [21, 22, 24, 30, 31, 33, 34], rehabilitation centres on an outpatient basis [16–20, 23, 26–29, 32, 36], and unspecified in two studies [25, 35]. In 16 studies an inclusion criteria was that patients were able to walk independently or had already regained the ability to walk, and only one study included non-independent walkers [24].

Five studies mentioned the use of walking assistive devices, four used walkers, canes or ankle–foot orthosis as an intervention if necessary [23, 30, 33, 35], and one compared the need for walking assistive devices before and after the intervention as an outcome [24].

All studies examined the effects of RAS and music-based RAS on gait and balance parameters after stroke. The interventions consisted of rhythmic auditory stimulation, using a metronome, music with marked beat, or real-time auditory stimulation feedback, in addition to another therapy as a conventional therapy, action observation training, treadmill or overground gait training, multidirectional step-up training, neurodevelopmental therapy, step-up training and multimodal Ronnie Gardiner Method. Control interventions consisted of conventional physical therapy, neurodevelopmental therapy, horse-riding therapy, multidirectional step-up training, action observation training, visual cueing, treadmill or overground gait training. Conventional physical therapy was inconsistently described and was not the same in all studies. Two studies mentioned that the intervention had no adverse effects [16, 24], two patients referred to pain, one in an affected upper limb [30] and one in a knee [25], the other studies did not inform about adverse effects.

Ten studies generated rhythmic auditory stimulation only via a metronome[19–22, 26, 27, 29, 32, 36, 37], five used music alone [16, 17, 23, 30, 35], three used a combination of a metronome and music [19, 24, 33], two used other stimuli such as an application or a software package [25, 34], and one used live music played by a music therapist [28]. In the studies with a metronome, a baseline assessment was performed to calculate the reach or step frequency. Afterwards, an increased or decreased auditory frequency was provided to examine the influence of the characteristics of the rhythmic sounds on the movement. These rhythmic sound sequences were generated via individual sounds generated by synthesizers or metronomes. In the studies with music, patients were exposed to different genres of music: classical wandering songs [23], melodies familiar to patients [33], commercial music of past and present musical genres. Only Gonzalez-Hoelling et al. informed about the specific pieces of music used in the intervention [24]. Song et al. did not specify the manner of sound production or apparatus used but did describe the motor task procedure, time of execution, and sound frequency in great detail [31]. Details of the intervention elements of all studies were extracted and are presented in Table 2.

**Table 2:** Details of the sound and music intervention elements

Type of metronome (*n* = 14)	Music used (*n* = 8)	Other stimuli (*n* = 3)
Cho et al., 2020 [19] Metronome application on the smartphone (Real Metronome, Gismart, United Kingdom). Via earphones	Elsner et al., 2019 [23]. Uniform MP3 music: classical wandering songs with a clearly accentuated beat. Via headphones	Yang et al., (2016) [34]. Microsoft visual C + + 2011 software with real-time auditive feedback
Lee et al., 2012 [37] Metronome (TU-88, BOSS, China). Via headphones (MDR-RF4000 K, Sony, Japan)	Wang et al., 2021 [33]. Music with melodies familiar to patients	Hutchinson et al., (2020) [25]. Progressive and Individualized Rhythm-Based Walking Training Program
Cha et al., 2014 [18] Metronome: no specifications	Bunketorp-Käll et al., 2019 [17] Music of Ronnie Gardiner Method™	Kobinata et al., (2016) [28]. A music therapist played steady beats using a musical rhythm instrument such as a drum or an autoharp
Wang et al., 2021 [33] Metronome: no specifications	Bunketorp-Käll et al., 2017 [16] Music of Ronnie Gardiner Method™	
Ko et al., 2016 [27] RAS smartphone application	Cha et al., 2014 [18] Specifically prepared music tapes with a synthesizer keyboard (KURZWEIL SP88, Young Chang Co., Ltd.) along with the MIDI Cuebase musical instrument digital interface program (Cubase MIDI Program, Steinberg, German), and a KM Player version 3.3 (KMP media Inc.)	
Lee et al., 2018 [29] Rhythmic auditory stimulation using digital audio editing software (GoldWave v5., GoldWave Inc., St. John’s, NL, Canada)	Mainka et al., 2018 [30] Functional training music was designed in accordance with the criteria described by Thaut (software cubase 3 SE). Via ear plugs through an ordinary MP3 player	
Cha et al., 2014 [19] Metronome: no specifications	Gonzalez-Hoelling et al., (2021) [24]. Music of past and present musical genres, with marked pulse, 1/4 or 6/8 rhythm, and variation of beats per minute. Via speaker: EasyAcc LX-839, 3W, 20 Hz–90 Hz, with Bluetooth and Micro SD	
Kobinata et al., 2016 [28] Metronome: no specifications	Collimore et al., (2023) [35]. Music with consistent beat saliency and rhythmic stability, and the playlist spanned various tempos and musical genres. Via headphones (AfterShokz AS451OB Sportz Titanium Open Ear Wired, Austin, TX, USA)	
Choi et al., 2021 [21] Metronome application on the smartphone (©2018 Soundbrenner)		
Gonzalez-Hoelling et al., 2021 [24] Metronome: Metronome & Tuner TGI 99B		
Kim et al., 2012 [26] Metronome application on the smartphone (ZyMi Metronome FREE). Via earphones		
Suh et al., 2014 [32] Digital Musical Instrument Digital Interface (MIDI) software. The rhythm stimulation was composed of single tone series in 4/4 time signature		
Chouhan et al., 2012 [22] Digital metronome software: no specifications		
Ahmed et al., 2023 [36] Metronome software program (Snapoh metronome). Via headphone (LGH-301 M.V)		

Outcome measures were predominantly related to gait and balance ability and spatiotemporal parameters of gait. Gait parameters included velocity, cadence, stride length, gait cycle duration and gait symmetry were often measured with a gait analysis system but some studies calculated these parameters after a 10 m walk test [17, 21, 23, 28, 31, 32, 35]. In order to assess gait ability, the studies used the Timed Up & Go Test [16, 24, 26, 29, 34], the 6 min walk test [17, 23], Functional Gait Assessment [21], Dynamic Gait Index [22, 26, 31], the Tinetti test [24], Functional Ambulation Category [24, 26] and the 3 min walking test [30, 36]. Balance ability and postural control were mostly assessed by the Berg Balance Scale [16, 19, 21, 23, 29, 33], but other scales such as Bäckstrand, Dahlberg and Liljenäs Balance Scale [16], Activities-specific Balance Confidence Scale [26] and instrumental evaluation with plates and specific software were also used [19–21, 29, 30, 32, 34]. Chouhan et al. (2012), Lee et al. (2018) and Wang et al. (2021) used the Fugl-Meyer motor assessment to measure motor control. Gonzalez-Hoelling et al. (2021) assessed functional independence with the Barthel Index and the Functional Independence Measure, and used the modified Rankin Scale and the NIHSS to assess stroke severity and disability. Collimore et al. (2023) not only assessed real-time gait analysis with a 18-camera motion analysis system at 200 Hz, but also oxygen consumption were collected on a breath-by-breath basis during each of the treadmill evaluation trials, as well as during the 4 min of quiet sitting that preceded each trial. Wang et al. (2021) measured the degree of treatment satisfaction with a Stroke rehabilitation treatment satisfaction questionnaire. Immediate post-intervention outcomes were always measured: short-term follow-up was measured in one trial (four weeks after the end of the intervention) [33], medium-term effects were reassessed in two trials three months after intervention [16, 23], and Bunketorp-Käll et al. (2017) did a follow-up at six months.

Functional ambulation ability, the use of walking assistive devices, functional independence, falls and quality of life were inconsistently reported. On the other hand, stroke hemisphere was reported but not analysed with regard to the intervention effect.

### Controlled trials

Fifteen studies stated that they were RCTs and one trial had a historical control group. Nine studies received a rating of a high risk of bias in the Cochrane Collaboration’s tool, but six of these studies received a rating of a low risk of bias in several domains, with the single exception of participant blinding (Supplementary Fig. 2). Bunketorp-Käll et al. (2017, 2019) was a high-quality study based on ratings of low risk of bias in many domains except for the blinding of participants, which is difficult in an RCT exercise.

Treatment in the intervention groups varied in intensity, frequency and duration and ranged from 30 min, 2 days a week for 3 weeks to up to 90 min, 5 days a week over 12 weeks. More detailed information about the intervention methods can be found in Table 3.

**Table 3:** Methods and interventions in controlled trials

Study	Research design	Treatment in intervention group	Treatment in control group	Intensity minutes, frequency duration
Ahmed et al., 2023 [36]	RCT	RAS (metronome) Treadmill training Physical Therapy	Treadmill training Physical Therapy	30 min/day 3 days / week 6 weeks
Bunketorp-Käll et al., 2017 [16]	RCT	Music-based therapy: Ronnie Gardiner Method *metronome* + music	2) Horse riding therapy 3) Control group, no therapy	90 min/day 2 days /week 12 weeks
Bunketorp-Käll et al., 2019 [17]	RCT	Music-based therapy: Ronnie Gardiner Method *metronome* + music	2) Horse riding therapy 3) Control group, no therapy	90 min/day 2 days /week 12 weeks
Cha Y. et al., 2014 [18]	RCT	Rhythmic auditory stimulation *metronome* + music	Intensive gait training	30 min/day 5 days/week 6 weeks
Cho et al., 2020 [20]	RCT	RAS (metronome) Action observation training Physical therapy	Action observation training Physical therapy	2 × 15 min/day 3 days/week 8 weeks
Choi et al., 2021 [21]	RCT	RAS (metronome) multi-directional step-up training	Multi-directional step-up training	30 min/day 3 days/week 4 weeks
Chouhan et al., 2012 [22]	RCT	Rhythmic auditory stimulation + conventional therapy	2) Visual cueing + conventional therapy 3) Conventional therapy	60 min/day 3 days/week 3 weeks
Elsner et al., 2019 [23]	RCT	Overground gait training Music-based RAS (classical wandering songs with a clearly accentuated beat)	Overground gait training	30 min 3 days/week 4 weeks
Gonzalez-Hoelling et al., 2021 [24]	NCT	Music-based RAS: RAS from NMT(*metronome* + music) Ronnie Gardiner Method	Conventional physical therapy	90 min/day 3 days/week 4﻿5.7 ± 20.6 days
Kim et al., 2012 [26]	RCT	Rhythmic auditory stimulation gait training (metronome) + neurodevelopmental training	Gait training + neurodevelopmental therapy	30 min/day 3 days/week 5 weeks
Lee et al., 2018 [29]	RCT	﻿Gait training with bilateral rhythmic auditory stimulation *metronome*	﻿Conventional rehabilitation with gait training without RAS (acoustic)	30 min/ day, 5 days/week 6 weeks
Mainka et al., 2018 [30]	RCT	Rhythmic auditory stimulation + treadmill training	1) Treadmill training 2) Neurodevelopmental therapy	15–17-20 min/day 5 days/week 4 weeks
Song et al., 2016 [31]	RCT	RAS + gait training + neurodevelopmental therapy	Gait training + neurodevelopmental therapy	30 min/day 5 days/week 4 weeks
Suh et al., 2014 [32]	RCT	RAS + gait training + neurodevelopmental therapy	Gait training + neurodevelopmental therapy	15 min 5 times/week 3 weeks
Wang Y. et al. 2021 [33]	RCT	Music therapy (1st metronome, 2nd music), drug therapy, rehabilitation training and walking training	Drug therapy, rehabilitation training and walking training	3 h/day 6 days/week 4 weeks
Yang et al., 2016 [34]	RCT	Real-time auditory stimulation feedback (RAF) + treadmill	Treadmill	30 min/day 3 days/week, 4 weeks

*RCT* randomized controlled trial, *NCT* No controlled trial; RAS: rhythmic auditory stimulation

Control groups did the same intensity, frequency and duration in all trials except in the research of Gonzalez-Hoelling et al. (2021), where the intervention group did 4 h and 30 min more of exercise per week than the control group. The treatment in the control groups was mostly conventional rehabilitation or gait training [19, 20, 23, 29, 34] but only three of the studies gave detailed information about the procedure in the control groups with conventional physical therapy [22, 24, 26, 33, 36]. Three control groups did neurodevelopmental treatment based on the Bobath concept [30–32], and the control group of Bunketrop-Käll did no therapy until finishing the study [16, 17]. The other control groups did the same intervention as the experimental group but without rhythmic auditory stimulation.

Nine of the trials reported improvements in the gait parameters (*p* < 0.05) in the intervention groups or significant between-group differences [19–21, 26, 31–34, 36], two studies did not obtain differences between the control and intervention groups [23, 24]. Three trials reported that the rhythmic auditory stimulation group improved more in their gait ability than the control groups, but the effects did not differ from the other intervention group [16, 17, 22]. In one trial, the rhythmic auditory stimulation group improved in their functional ambulation ability and needed less walking assistive devices at discharge compared to the control group [24]. In three trials the improvements were dependent on different factors such as the measure used, the studied outcomes and the size of the stroke [24, 26, 30] (Table 4). Given the heterogeneity of the interventions and the varied durations of the 8 RCTs, it did not seem appropriate to pool the results for the purposes of a meta-analysis.

**Table 4:** Outcomes, measures and main results

Study	Outcomes of interest	Measures	Main results
Ahmed et al., 2023 [36]	gait velocity step cycle step length	3-min walking test (3MWT)	Both groups increased post-treatment walking speed, step cycle, step length, percentage of time on each foot and ambulation index Improvement in gait parameters was significantly higher in the study group compared to the controls (*p* < 0.05)
Bunketorp-Käll et al., 2017 [16]	gait ability balance	Timed Up&Go Berg Balance Scale Bäckstrand, Dahlberg and Liljenäs Balance Scale (BDL-BS)	Differences between music-based therapy and control group No differences between music-based therapy and horse-riding therapy Music-based therapy improved balance more after 6 months
Bunketorp-Käll et al., 2019 [19]	gait capacity gait speed	10-m walk test 6 min walk test	Differences between music-based therapy and control group in gait capacity No differences between baseline and reassessment with music-based therapy in gait speed Significant improvement at 6-month follow up for gait capacity in the horse-riding therapy compared to control group
Cha H. et al., 2014 [19]	gait velocity cadence stride length on the ispilesional side stride length on the contralesional side gait symmetry	GAITRite system	Gait velocity, cadence, and stride length on the contralesional side were significantly decreased under the RAS − 10% conditions Gait velocity and cadence were significantly improved, but gait symmetry was significantly decreased under the RAS + 10% and + 20% conditions compared with under the RAS 0% conditions
Cha Y. et al., 2014 [18]	gait performance postural control	GAITRite Berg Balance Scale multifunction force measuring plate	Significant improvement in the RAS group for gait performance and postural control compared to control
Cho et al., 2020 [20]	static balance dynamic balance	Biodex Balance System	Significant improvements in both groups for static and dynamic balance, but a greater degree of changes were observed in the action-observation training group than those in the control group
Choi et al., 2021 [21]	gait ability static balance ability dynamic balance ability	Functional Gait Assessment 10 m walk test Berg Balance Scale Balancia software (velocity, path length and sway area)	RAS group showed a greater difference in the amount of change in every gait and balance ability assessment compared to the control group
Chouhan et al., 2012 [22]	dynamic balance upper extremity motor control	Dynamic gait index Fugl Meyer motor assessment	RAS improved gross motor, fine motor, and gait imbalance more than conventional group RAS and visual cueing were equally effective in improving gross motor, fine motor, and gait imbalance
Collimore et al., 2023 [35]	step time stance time swing time gait symmetry energetic cost of walking	Real-time gait analysis ﻿with a 18-camera motion analysis system at 200 Hz ﻿Oxygen consumption (VO2) data were collected on a breath-by-breath basis (Cosmed© K5, Rome, Italy)	Post-treatment reductions in step time, stance time and swing time asymmetries were observed. A reduction in the energetic cost of walking was obtained and detected to be strongly dependent on the degree of baseline energetic impairment
Elsner et al., 2019 [23]	walking capacity walking velocity stride length balance	6 min walk test 10 m walk test Berg Balance Test	RAS and control group did not differ significantly on walking capacity, walking velocity, stride length or balance
Gonzalez-Hoelling et al., 2021 [24]	gait and balance parameters trunk control walking ability functional independence stroke severity and disability	Timed Up& Go Test Tinetti Test Functional Ambulation Category assistive devices Functional Independence Measure Barthel Index modified Rankin Scale National Institutes of Health Stroke Scale (NIHSS)	﻿No between-group differences were identified for gait and balance parameters nor for secondary outcomes Music-based RAS improved in the Functional Ambulation Category more than the control group
Hutchinson et al., 2020 [25]	safety feasibility walking speed walking cadence	10 m walk test optical motion capture system (Qualisys AB)	Safety was reported with no falls Increases in walking speed and walking cadence
Kim et al., 2012 [26]	dynamic balance gait ability spatiotemporal parameters of gait	Activities-specific Balance Confidence (ABC) Scale, Dynamic Gait Index (DGI), Four Square Step Test (FSST), Functional Ambulation Category (FAC), Timed Up&Go test (TUG test) Up stair and Down stair times GAITRite system	Increases in dynamic balance and spatiotemporal gait parameters were observed in both groups Compared with the control group, the RAS group showed significant improvements in scores on the ABC scale, DGI, TUG, and Up stair and Down stair times
Ko et al., 2016 [27]	cadence speed stride length gait cycle duration step length	G-Walk GAITRite system	After gait training with rhythmic auditory stimulation, gait speed, cadence, stride length, gait cycle duration, and step length of the contra and ipsilesional sides improved significantly compared to baseline
Kobinata et al., 2016 [28]	gait cadence velocity stride length gait balance	10 m walk test	Pre- versus post-test measures revealed significant increases in velocity and stride length in the cerebellum, pons and medulla, and thalamus groups
Lee et al., 2018 [29]	﻿gait symmetry gait ability balance ability lower extremity function	﻿gait analysis system (OptoGait) Timed Up&Go test (TUG) Berg Balance Scale (BBS) Fugl-Meyer Assessment (FMA)	﻿Gait symmetry on step time significantly improved more in the gait training with bilateral rhythmic auditory stimulation group than in the control group The gait training with bilateral rhythmic auditory stimulation group showed significantly greater improvement in gait ability than the control group Both groups showed significant improvements, but did not differ significantly, in the Timed Up&Go test (TUG), Berg Balance Scale (BBS), and Fugl–Meyer Assessment (FMA) compared to baseline
Mainka et al., 2018 [30]	gait velocity cadence stride length gait symmetry endurance postural stability	﻿fast gait speed test (FGS) locometre (LOC) 3-min walking test (3MWT) instrumental evaluation of balance (IEB)	﻿Significant group differences in the FGS for adjusted post-measures in gait velocity and cadence Stride length results did not vary between the groups. LOC, 3MWT, and IEB did not indicate group differences
Song et al., 2016 [31]	Gait ability cadence step length functional gait ability	10 m walk test GAITRite analysis system Dynamic Gait Index	Both groups improved, but RAS group showed more significant increase in cadence, step length, 10 m walk test, and dynamic gait index
Suh et al., 2014 [32]	gait velocity stride length cadence standing balance	10 m walk test Biodex balance system (BBS) of Biosway®	Significant improvement in RAS group for gait velocity, stride length, cadence, overall stability index, mediolateral index and anteroposterior index over the control group
Wang Y et al., 2021 [33]	walking ability lower extremity motor function balance ability degree of treatment satisfaction	FreeStep gait analyzer Fugl-Meyer Assessment (FMA) Berg Balance Scale (BBS) Stroke rehabilitation treatment satisfaction questionnaire	Stride length, cadence and maximum velocity were higher in the music therapy group compared to the control group The difference in step length between the contralesional side and healthy side was significantly lower in the music therapy group than in the control group The FMA and BBS scores were significantly higher in the study group than in the control group The music therapy group had a significantly higher satisfaction rate than the control group
Yang et al., 2016 [34]	static balance dynamic balance and gait ability spatiotemporal gait parameters	Blancia (wii Balance board) Timed Up&Go Test GAITRite system	Significant differences in static balance and the Timed Up&Go Test were observed in the Real-time auditory stimulation feedback group compared with the control group The real-time auditory stimulation feedback group showed significant improvements in gait speed, step length and stride length, single limb support percentage of the contralesional side, and gait asymmetry compared with the control group

### Non-controlled studies (pre-post studies)

Five of the included studies were pre-post intervention designs and all reported significant within-group differences in measures [18, 25, 27, 28, 35]. All of these studies received a rating of high risk, mainly because of the lack of a control group and the results were with no or very limited follow-up. The studies varied in terms of the type of intervention, from walking in different speed conditions to walking in different input conditions. The duration of the interventions ranged from 5 to 30 min (Table 5).

**Table 5:** Methods and intervention in non-controlled studies

Study	Research design	Intervention treatment	Intensity minutes, frequency, duration
Cha et al., 2014 [18]	Pre-post study	walking in 5 conditions: 1) no RAS 2) baseline-matched RAS 3) -10% 4) + 10% 5) + 20% *metronome*	Duration depending on the time needed to walk the distance of 457 cm 3 times
Collimore et al., 2023 [35]	Pre-post study	Automated gait rehabilitation delivered via the closed-loop control of music	30 min
Hutchinson et al., 2020 [25]	Pre-post study	Progressive and individualized rhythm-based walking training programme	30 min
Ko et al., 2016 [27]	Pre-post study	walking in 5 conditions: 1) no Ras 2) baseline-matched RAS 3) -10% 4) + 10% 5) + 20% with *metronome*	﻿Each RAS condition was practiced for 10 min. A 3-min adaptation period and a 7-min gait-training period were included
Kobinata et al., 2016 [28]	Pre-post study	Rhythmic auditory stimulation *metronome* or musical rhythm instrument (drum or autoharp)	20 min

*RAS* Rhythmic auditory stimulation

## Discussion

Our systematic review of the literature suggests that the available evidence regarding the effects of rhythmic auditory stimulation on functional ambulation ability in stroke patients is heterogeneous and largely of low quality and that the trials report some benefit compared to no intervention prescription or conventional rehabilitation, (physical therapy, neurodevelopment therapy, etc.), a finding that is consistent with previous narrative reviews [7, 11, 12, 38–40]. Functional ambulation ability was mostly assessed with spatiotemporal parameters without taking into account the need for human assistance or walking assistive devices to be able to walk. The use of walking assistive devices and the lesioned area was inconsistently described, making it impossible to draw comparisons between the intervention effects or analyse their relationship with improvement. The stroke type was either ischaemic or haemorrhagic, but no study undertook a subgroup analysis to find out if one stroke type had significantly greater improvements in results than the other.

Rhythmic auditory stimulation would seem to add value to the effect of an intervention on gait parameters, coinciding with the revision of Wang et al. (2022). However, when comparing a group who did rhythmic auditory stimulation with a group who did another therapy or cueing (horse therapy and visual cueing), between-group differences are not always apparent. As is seen in a recent scoping review by Saraiva et al. (2023), each method or therapy gives unique benefits that can help to improve stroke survival. Furthermore, the importance of multidisciplinarity needs to be taken into account. This approach, which is ever more present in stroke rehabilitation, favours neuroplasticity and improves motivation towards therapy [41].

The risk of bias of the three trials reporting no differences in between-group results was categorised as high and the quality as low, suggesting that these findings may not be generalizable.

With regard to sample sizes and characteristics, the trials had small samples from 11 to 123 people. Randomization was ensured in most of the clinical trials but only assessment blinding was possible, although some studies either did not perform this blinding or did not inform about this question.

In Kobinata et al. (2016), significant increases in velocity and stride length in the cerebellum, pons and medulla, and thalamus groups were found, but although the putamen and corona radiata groups showed increases, these were not significant. No other study performed the subgroup analysis of the different brain areas, possibly because sample sizes were too small. For instance, Gonzalez-Hoelling et al. have recently published a second analysis of the study included in this review in which they compared gains by brain areas. It would be interesting for clinical trials to consider the affected brain area as an outcome for subgroup analysis of the effect of rhythmic auditory stimulation as this might help in being able to predict the rehabilitation effect of rhythmic auditory stimulation by affected areas. Cerebellar stroke patients should also be considered because the cerebellum is responsible for the prediction of sensory consequences of movement and perform as an orchestrator of motor commands and the explicit production of state changes. The lesioned hemisphere was collected but no study performed a subgroup analysis about the differences in improvement. It is likely that no significant differences between right or left hemisphere will be found when we consider that rhythm is processed in both hemispheres [7, 42].

The stroke phases were generally chronic with only one third of the studies including subacute patients. It may be difficult to ensure the follow-up of patients included in hospital settings, and in acute and subacute phases the prognosis might be less sure than in a chronic phase. Compared to the chronic stroke patients that significantly improved gait after rhythmic auditory stimulation, subacute stroke patients have more divergent results. Although subacute patients are found to improve with respect to admission, the dimension of their improvement does not seem to vary from that of other stimulation treatments. We agree with other reviews that the spontaneous recovery component must also be considered, which occurs up to about six months after stroke onset [38].

It was difficult to compare the interventions considering the heterogeneity of rhythmic or music stimulation used. The name of rhythmic auditory stimulation itself was not applied with a single sense and procedures varied from trial to trial as were the pieces of music that were used. Since control treatments varied in each trial, it was not possible to do either a subgroup analysis or a meta-analysis. Only three studies reported using a professional music therapist [24, 28, 30]. Other than this, two interventions were conducted by a physiotherapist [23, 29], but most reports simply failed to provide this information. Despite agreeing with Magee et al. (2017) that interventions result in greater improvements when conducted by trained music therapists [12], the absence of data here does not allow us to draw this conclusion from our results. Poor study design, with no professional therapist, and the lack of reporting about control treatments across many trials weaken the conclusions. Even so, it is evident, and all the trials coincide in this, that patients who receive rhythmic auditory stimulation significantly improve at the end of the intervention in comparison with their baseline state. Neither the clinical trials nor the pre-post studies were consistent in the length of sessions and the duration of the interventions. There is still no consensus as to whether the length of the RAS intervention is related to the amount of effect.

Most of the studies that specified this aspect included people who were already walking autonomously as inclusion criteria. If the patients had to be able to walk autonomously, they would probably be grouped in the highest functional ambulation category, without the possibility of improvement, so making it impossible to see the differences between the beginning and end of treatment. Gonzalez-Hoelling et al. (2021), included patients who were not walking independently and who had low motor function [24]. Perhaps for this reason, and as is also suggested by Janzen et al. (2022) [7], the effectiveness of the intervention in the trial of Gonzalez-Hoelling et al. (2021) was limited due to the capacity of motor function at enrolment. It should also be noted that it is possible that the use of walking assistive devices may be governed in part by patients’ perception of their walking ability, as detected by Tyson and Togerson (2009) [5], rather than being a genuine indicator of improvement. Some studies consider the use of walking assistive devices itself as a treatment and, as is seen in the systematic review of Hugues et al. (2019), is an effective intervention for balance [43]. Even so, we recommend that more studies be carried out including non-walkers at baseline and that the use of walking assistive devices be considered as an outcome.

Although there are studies that examine neuroplasticity and the effect of music and rhythm on brain areas [44–49], evidence that compares the degree of effect of a rhythmic auditory stimulation intervention depending on the lesioned area is scarce [50]. We suggest that more research about the relationship between the effect of a rhythmic auditory stimulation intervention and the lesion area is necessary to improve the generalizability of the results to the stroke population.

In seven studies, the patients undertook rehabilitation in a hospital on an in-patient basis, and eleven studies were with community-dwelling patients. Although the results of community-based studies are generally more applicable to the external population [11], stroke patients in the subacute phase are usually in hospital whereas outpatients are normally in a chronic phase, making it difficult to compare the two populations.

There are a number of methods for assessing walking and no clear consensus on which should be used in clinical trials for assessing functional ambulation or walking ability; however, it has been suggested that the Timed Up & Go test may be favoured because it is easy to measure, although we agree with Hafsteinsdóttir (2014) that it is only recommended after stroke in patients who are able to walk. Only categorical measures (Functional Independence Measure, Barthel Index, Functional Ambulation Category) can capture both non-walkers as well as walkers or wheelchair users. An assessment consensus should be found for functional ambulation ability and the use of assistive devices in stroke, as in the case of spinal cord injury with the Spinal Cord Injury Functional Ambulation Inventory [51].

### Study limitations

One limitation of the present review is that the heterogeneity across the music-based or rhythmic auditory stimulation used in the studies, session intensities and frequencies, as well as the different outcome measures used, small sample sizes, and lack of available data related to the studies’ outcomes, do not allow for a meta-analysis to be undertaken at this time. Therefore, we strongly recommend that future studies should provide more detailed description of participants and that validated outcome measures be adopted as standard.

Considering the limitations in the quality of most reviewed studies (low to moderate) and the high risk of bias due to the lack of clarification on the procedures for assigning the participants to the different interventions, these findings need to be interpreted with caution.

Finally, the external validity of the trials to date may be limited as, for example, few studies included stroke patients in a subacute phase, many excluded non-walkers, and most were single-centre trials.

## Conclusion

There is a significant level of diversity in the studies investigating the effects of rhythmic auditory stimulation and music-based rhythmic auditory stimulation in stroke patients, and the available evidence on their clinical benefit is in general inconclusive. The best available evidence suggests that functional ambulation ability of chronic stroke patients improves after an intervention with music or rhythmic auditory stimulation and that the gains are greater than with no treatment or conventional therapy. The use of walking assistive devices remains a possible functional outcome to be researched in the future. The improvements with RAS or music-based RAS in relation to the lesioned area are still uncertain.

Future clinical trials should include people of all stroke phases with a broad range of stroke lesion areas, for both short-term and long-term outcomes, and analyse the effect of rhythmic auditory stimulation in relation to the lesioned area. We recognize the need for consensus on a paradigm for music-based and rhythmic auditory stimulation and for good quality studies.

### Supplementary Information

**Additional file 1: Table 1. **Search strategies developed for electronic database. **Table 2. **PRISMA Checklist. **Figure 1. ﻿**Risk of bias graph: review authors' judgements about each risk of bias item presented as percentages across all included studies. **Table 3. **Quality assessment PEDro, controlled trials. **Figure 2. **Risk of bias rating in the Cochrane Collaboration’s tool.

## Data Availability

All data generated or analysed during this study are included in this published article [and its supplementary information files].

## Abbreviations

RAS: Rhythmic auditory stimulation
RCT: Randomized controlled trials
